# Replication Crisis in Psychology, Second-Order Cybernetics, and Transactional Causality: from Experimental Psychology to Applied Psychological Practice

**DOI:** 10.1007/s12124-024-09867-3

**Published:** 2025-01-08

**Authors:** Alexios Brailas

**Affiliations:** 1 Department of Psychology, Panteion University, Athens, Greece; 2 Athenian Institute of Anthropos, Athens, Greece

**Keywords:** Methodology, Epistemology, Replication crisis, Second-order cybernetics, Causality, Psychological practice

## Abstract

This article aims to reconceptualize the replication crisis as not merely a problem of flawed methods, lack of scientific rigor, or questionable researcher conduct, but as a fundamentally epistemological and philosophical issue. While improved methodologies and scientific practices are necessary, they must be considered through the lens of the underlying epistemologies. Toward this end, a new paradigm for psychological research and practice, grounded in second-order cybernetics and transactional causality, is proposed as instrumental. Second-order cybernetics, as introduced by Heinz von Foerster, challenges traditional scientific methodologies that assume a strict separation between the observer and the observed. The core idea is that the observer, through the very act of observing, inevitably becomes part of the system they study, leading to a shift from linear to transactional causality. This epistemological shift has profound implications for the research practice and the responsibility of the psychology practitioner. Foerster’s ethical imperative –*act always so as to increase the number of choices*– combined with the aesthetic imperative –*If you desire to see*,* learn how to act*– illuminates an alternative methodological landscape for the clinical practice. The replication crisis in psychology is examined in light of these theoretical shifts, allowing for a new constructive vision which integrates basic research with applied psychological practice. Second-order cybernetics encourages a participatory approach to research, emphasizing the catalyzing role of the observing practitioner. The article concludes by advocating for an epistemological superposition, where psychologists navigate multiple perspectives to enhance the integrity and applicability of their findings in the real world.

## Introduction

In this article, I aim to offer an alternative perspective on the issue of reproducibility in psychological research (Open Science Collaboration, 2015) through the lens of second-order cybernetics and transactional causality (Von Foerster, 2003). I begin with a brief introduction to the replication crisis in psychology, including the underlying assumptions, to provide the context. Next, I introduce second-order cybernetics and transactional causality as an epistemological framework. I also discuss the implications of this framework for addressing the challenge of reproducibility. Finally, I analyze a contentious research study that unfolded alongside the replication crisis in psychology. Drawing on second-order cybernetics, I present and explore an alternative epistemological perspective on this case and I discuss the implications for psychological research and practice.

The purpose of this article is not to discuss the replication crisis or how to address it through methodological modifications and reforms within the positivist research paradigm, something that is already extensively covered in the existing literature (Munafò et al., 2017). Instead, my aim is to explore the replication crisis from a different epistemological perspective: What if the observing researcher cannot, in principle, be detached from the system they observe/ study? This epistemological shift has significant implications for psychological practice, both in social and clinical settings, as it allows participants in interventions to realize alternative and more enacting futures for themselves and their communities.

The methodological framework for this study is grounded on second-order cybernetics and particularly on the *ethical* and the *aesthetic* imperatives as were formulated by Von Foerster (2003). By applying these two principles to the “power pose” hypothesis (Carney et al., 2010), a theory based on a study that many scholars have failed to replicate (Ranehill et al., 2015), I demonstrate how even a contentious study can serve as a valuable tool for the informed psychology practitioner working in naturalistic, real-world contexts.

As the observer is always included in the system within the field of observation and cannot escape it, a critical question that arises is in what ways can we incorporate this perspective in our research methods in a constructive way? To this end, this article concludes by discussing the importance of methodological eclecticism, epistemological awareness and literacy, research flexibility, and methodological diversity, as ways to further enrich and advance psychological research practice.

## The Replication Crisis in Psychology

In 2005, John Ioannidis, in his landmark paper titled *Why Most Published Research Findings Are False*, critically examined the reliability of scientific research, focusing on the field of biomedicine. Likewise, a replication of 100 experimental and correlational studies was attempted to assess the reproducibility of research findings in psychology. Only about 36% of the replicated studies produced statistically significant results similar to the original findings, with effect sizes that were, on average, of half the magnitude (Open Science Collaboration, 2015).

Ioannidis (2005) argued that a significant proportion of published research findings did not replicate due to a combination of methodological factors and widespread scholarly practices in academia –such as the overreliance on p-values and replication bias. And today, there is an ever-growing body of literature discussing the phenomenon of hidden flexibility, like p-hacking, where researchers effectively manipulate data until they obtain significant results, for instance, a p-value just below the conventional threshold of 0.05 (Chambers, 2017; Ioannidis, 2005).

Such manipulation often involves multiple, unreported analyses, research designs with low statistical power, or the tweaking of study parameters, such as selectively excluding outliers, choosing specific covariates, or stopping data collection at convenient times to achieve a desired result (Chambers, 2017; Head et al., 2015; Ioannidis, 2005; Munafò et al., 2017; Simmons et al., 2011; Wicherts et al., 2016). Although these practices may not be overtly fraudulent and often occur with good intentions or out of ignorance, they nonetheless create a distorted scientific literature filled with unreliable findings (Chambers, 2017).

Many scholars point out that the challenges to reproducibility are often systemic and culturally rooted in the pressures researchers face to publish in prestigious journals, secure funding, and advance their careers, the famous “publish or perish” dogma (Chambers, 2017; Munafò et al., 2017). Implementing reforms such as preregistration, open data, and transparency initiatives is believed to improve reproducibility and credibility in psychological science (Chambers, 2017; Munafò et al., 2017; Simmons et al., 2011). However, the replication crisis is deeply rooted in both methodological and epistemological issues regarding the nature of scientific inquiry (Earp & Trafimow, 2015; Park, 2020) which deserve more discussion and careful consideration.

### The Epistemological and Methodological Assumptions Implied

Reproducibility is considered a foundational principle of scientific methodology in classical sciences like physics or chemistry, where experiments must be repeatable under the same conditions, yielding identical results again and again regardless of the experimenter (Atmanspacher & Jahn, 2003). This positivist research paradigm became dominant in psychology and many other social disciplines during the 20th century, in an attempt to distance themselves from philosophy and acquire the status and prestige of the classical sciences (Dafermos, 2014, 2021). Within psychology, the notion of reproducibility relies on the hypothesis that psychological phenomena are assumed to be generalizable across different contexts and time. Failure to replicate then is typically interpreted as an issue with the original findings, rather than as a result of contextual differences.

However, due to the complexity of human behavior and the numerous variables that can influence experimental outcomes, it is often practically difficult to determine whether a failure to replicate is the result of methodological differences, chance, or unrecognized and unaccounted-for factors (Earp & Trafimow, 2015). Flis (2019) critiques the narrowed response to the replication crisis in psychology, which reduces the problem to a set of psychological flaws in individual researchers, overlooking the complexities of how science as a cultural system per se produces and legitimizes knowledge. Experiments and their replications are shaped by conceptual and material assumptions, which in many cases make exact replication impossible (Feest, 2019). Differences in sample populations, cultural contexts, and minor methodological variations can significantly impact the ability to replicate findings. Reproducibility could be unachievable when human participants are aware of experiment repetitions (something that is often unavoidable, as participants are embedded within an ever-changing cultural landscape), thereby altering the conditions themselves by creating a looping effect: “A causal understanding, if known by those who are understood, can change their character, can change the kind of people that they are. That can lead to a change in the causal understanding itself.” (Hacking, 1996, p. 351). These challenges are particularly problematic in psychology and behavioral sciences, where the subjective and evolving nature of human participants complicates further the reproducibility of experiments (Atmanspacher & Jahn, 2003). Therefore, while replication is essential in the positivist research paradigm (mostly in quantitative studies), it must be approached with an epistemic awareness of the inherent limitations and complexities in psychological research. Teo (2020) critiques psychology’s ‘hyper-scientific’ approach, where the discipline artificially inflates its methodological rigor to mask its lack of a natural science foundation. In this article, I argue for an epistemological shift to address the issue of replication failures in a new, more enactive and enabling way.

## Observers as Parts of the System they Observe: Transactional Causality and Second Order Cybernetics

Cybernetics emerged as a transdisciplinary field in the 1940s focusing on the study of communication and control in both living systems and machines (Scott, 2004). The term was coined by Norbert Wiener, inspired by the Greek word *Κυβερνήτης* (Wiener, 1948/2019). The Macy Conferences on Cybernetics, which took place between 1946 and 1953, played a pivotal role in shaping the field (Umpleby, 2016). Cybernetics places particular emphasis on the concept of the feedback loop: every result feeds back to its cause. Therefore, every process should be realized as a cyclical one. This idea may seem quite simple, but its implications are dramatic, especially in combination with the concept of a system. A system can be broadly defined as a set of interdependent and interrelated parts with emergent properties that differ from those of the individual components (Kauffman, 1996). As a result, the traditional linear way of interpreting phenomena was challenged.

Traditional cybernetics theory, now referred to as first-order cybernetics, primarily focuses on observed systems, that is how systems operate, maintain stability, and achieve goals through feedback mechanisms and loops, as an independent observer perceives them. Heinz von Foerster was a key figure in the development of second-order cybernetics, which he briefly defined as the cybernetics of cybernetics (Scott, 2004; Von Foerster, 2003). Second-order cybernetics introduced the concept of observing circularity, where the observer is no longer detached or isolated from the system but is instead considered a participating actor in the loop. This inclusion of the observer fundamentally alters the nature of observation, making it a self-referential process. The observer feeds back into the system, and the system, in turn, feeds back into the observer, creating a kind of transactional causality. In other words, second-order cybernetics requires us to consider how our observations and actions influence the very systems we study. This shift has profound implications for scientific methodology, extending beyond the realms of cybernetics and systems science (Umpleby, 2016).

With second-order cybernetics, Von Foerster challenged the traditional scientific methodology, which required the separation of the observer from the observed to ensure objectivity. He argued that this principle is not only flawed but also impossible to achieve, as it ignores the inherent transactional influence of the observer on the observed system (Von Foerster, 2003). The denial of this influence, he suggested, is rooted in a fear of the inevitable paradoxes that arise when the observer is recognized as an active part of the system they observe:

What appears to us today as being most natural to see and think, was then not only difficult to see, but wasn’t even allowed to be thought. Why? Because it would violate the basic principle of scientific discourse which demands the separation of the observer from the observed. It is the principle of objectivity. The properties of the observer shall not enter the description of his observations. (Von Foerster, 2003, p. 287)

In traditional linear causality, the relationship between cause and effect is viewed as unidirectional: a single cause leads to a single effect. Transactional causality, however, recognizes that causation is reciprocal, involving concurrent interactions among the elements within a system. This shift is what Gergen later referred to as a departure from influence to confluence (Gergen, 2009). The concept of transactional causality is wonderfully visualized in M.C. Escher’s famous lithograph the *Drawing Hands*, where one hand appears to draw the other at the very moment that it is drawn into existence by the other. Transactional causality can be traced back to the theories of Heraclitus and Cratylus:

Heraclitus argued that no person can ever step into the same river twice, because in every attempt they would be a different person in a different river. Cratylus, a student of Heraclitus, took this idea further: A person cannot step into the same river even once, as both the person and the river change because of the act of stepping in. (Brailas, 2024, p. 510)

Transactional causality implies that all participants in a research study (the researchers included) as well as their cultural and historical context are all in a process of concurrent reciprocal determination: “We call this approach ‘transactional’ because it fosters the free multilateral transaction of all those taking part. … The approach is at the same time analytic and interrelational, as we feel that one cannot be fully developed without the other.” (Vassiliou, 1968, p. 65) The observations will always return to the observer, forming a closed loop of interdependence, and the whole system cannot be fully understood without acknowledging the observer’s role in shaping its reality (Archibald Wheeler, 1977).

## Replication Crisis and the Need for a New Epistemology

If the observer is part of the system they observe, how can we ever speak of replication? It is not only because participants in a study would differ. Replication is not possible in principle within a second-order cybernetics epistemology because different researchers inherently differ; they have different intentions. The researcher of the initial experimental study may believe in an idea and seek to prove their hypothesis correct. The researcher of the replication study may, on the other hand, doubt the original hypothesis and aim to disprove it. In the first instance, a researcher gains recognition by proving something right, while in the second, a researcher gains recognition by proving something wrong. As Sexton (1997) points out, “The perspective of the observer and the object of observation are inseparable; the nature of meaning is relative; phenomena are context-based; and the process of knowledge and understanding is social, inductive, hermeneutical, and qualitative.” (p. 8).

And what about double-blind research designs? Don’t they ensure objectivity by separating the observer from the observed? Only to some extent. The observer is always a part of the system observing the other parts. The slightest changes or perturbations in one part of the system can lead to complex and unpredictable outcomes in the long run (butterfly effect) (Mitchell, 2009). When we move from classical to social sciences, the feasibility of conducting reproducible experiments becomes much more uncertain (Atmanspacher & Jahn, 2003). This uncertainty arises because, in studies involving human behavior, both participants and researchers can be aware that the experiment is being replicated. This self-awareness, or reflexivity, fundamentally alters the original conditions under which the experiment was conducted. The knowledge that they are part of a replication study, along with awareness of previous results, can influence participants’ responses, thus modifying key variables and changing the experimental environment. As a result, recreating the exact conditions of the original experiment becomes impossible, making true replication difficult, if not impossible, in these fields. The reflexivity of human subjects introduces an inherent variability that undermines the ability to achieve identical conditions in replicated studies (Atmanspacher & Jahn, 2003).

Reproducibility in the social sciences demands much more sophisticated approaches than those typically used in traditional physical sciences. We need to develop models of psychological inquiry that account for this complex interdependence between human actors and the material world (Atmanspacher & Jahn, 2003; Von Foerster, 2003). These models should be better equipped to handle the inherent complexity of these systems and offer more complex ways to judge their validity.

## A Case Study: The Power Posing Debate

Let’s consider now the following case of a contentious research study that unfolded in parallel with the replication crisis in psychology. I will then attempt to provide an alternative epistemological perspective on this case through the lens of second-order cybernetics.

In their controversial experimental study, Carney et al. (2010) explored how adopting high-power or low-power nonverbal poses for just two minutes could influence physiological and psychological states. Their findings suggested that brief postural changes could produce measurable hormonal and behavioral changes, supporting the embodiment of power and its potential real-world implications.

Based on these findings, one of the authors of the study, Amy Cuddy delivered a TED Talk titled “Your body language may shape who you are”. The talk soon became viral with millions of views worldwide. Her talk emphasized the practical implications of power posing, inspiring individuals to harness body language to build confidence and resilience in their daily lives. However, as the replication crisis in psychology emerged in the 2010s, this study became a focal point of contention (Dominus, 2017).

Ranehill et al. (2015) conducted a conceptual replication of Carney et al.’s power-posing study with a significantly larger sample size of 200 participants and a more rigorous research design. While the study confirmed that power poses significantly increased self-reported feelings of power, it found no significant effects on hormonal levels or risk-taking behavior, raising questions about the integrity of the original study.

Soon, a ‘scientific war’ unfolded, with many methodologists driving the replication crisis movement involved, and many accusations of ‘methodological terrorism’, scientific bullying, or research malpractices being expressed by both sides (Dominus, 2017; Gelman & Fung, 2016). Numerous empirical and methodological studies emerged on the subject, with some partially confirming power posing theory and others refuting it (Loncar, 2021). Amid the controversy, Carney, the first author of the original study, ultimately renounced the power posing theory^1^ while Cuddy continued to support it, citing a growing body of literature on the subject (Cuddy et al., 2018).

## Epistemological Superposition: The Road from Experimental Psychology to Applied Psychological Practice

In quantum physics, the famous Schrödinger’s cat, enclosed in a box, is neither alive nor dead but exists in a superposition of states until someone opens the box. At that point, the cat collapses into one of two distinct states, alive or dead, a binary yes/no condition. Similarly, in the ancient oracle of Pythia, the cryptic phrase “You will go, you will return never in the war you will perish” lacks a definite meaning until someone decides where to place the comma.

The power poses hypothesis emerged and coevolved with the growing awareness of the reproducibility crisis in psychology. Loncar (2021) provides a concise overview of the empirical evidence on the subject. But the question remains: to power pose or not to power pose? This is a valid question only within a first-order cybernetics epistemology, where an objective observer exists, and a yes/no answer should be provided through rigorous scientific inquiry. The replication crisis and the methodological movement that followed have undoubtedly improved scientific practices, such as promoting open science, pre-registering studies, and acknowledging the importance of publishing replications. Journals now also accept negative results more readily. The use of methodological tools like meta-analyses and p-curving diagrams has expanded, and researchers have developed greater methodological literacy, with concepts like p-hacking becoming well understood. However, the epistemology behind the methodology is a core part of scientific inquiry we should focus on.

So, should we power pose or not? Without neglecting the need for sound and robust scientific practices, within a second-order cybernetics epistemology, the answer need not be a binary yes or no. It can exist in a superposition of states, depending on the observer and the participating actors. Power poses may work or may not, depending on the specific situation. In first-order cybernetics, the researcher is considered to be an objective observer of reality. In second-order cybernetics, the researcher is an active participant, a catalyst in the unfolding of reality that inevitably facilitates transformations (Cabell & Valsiner, 2014). Psychological processes unfold over time in a manner that is inherently linked to the specific contextual parameters and future-oriented goals of the participating actors and of the whole they co-create with their synergies (Agazarian, 1992; Valsiner, 2017). The ancient oracle could answer the question by implying a superposition of outcomes: *You will power pose*,* you will elevate yourself never in the world you will perish.*

### Act so as to Increase the Number of Choices

Each psychological phenomenon, embedded in its unique social and historical context, necessitates tailored methodological approaches (Valsiner, 2017). Recognizing the observer as an integral part of the system means they bear greater responsibility for their actions. This realization led to Foerster’s famous *ethical imperative*: “Act always so as to increase the number of choices” (Von Foerster, 1984, p. 60) Placing the observer within the observed system implies that meaning is constructed collaboratively rather than imposed unilaterally. In this sense, scientific inquiry becomes a practice of creating shared understanding and expanding the space for mutual interaction, both of which are deeply ethical activities.

A practitioner working with human systems and being an expert in facilitating action research projects might decide to utilize power poses in a community intervention, alongside other tools for empowering people. They would test in practice whether power poses are realized as meaningful and beneficial in their situated action. Adhering to a reproducibility-bounded, yes/no epistemology, you don’t have to decide anything, you don’t have to take the responsibility to develop a customized action plan. You cannot undertake the responsibility to influence your future through your actions. Why can’t you? “Simply because the decidable questions are already decided by the choice of the framework in which they are asked… ultimately we arrive after a long sequence of compelling logical steps at an irrefutable answer; a definite ‘yes,’ or a definite ‘no.’” (Von Foerster, 2003, p. 293).

Von Foerster (2003) argued that the freedom to choose comes with the responsibility to make decisions that enhance, rather than limit, the offered choices. Adhering to a second-order cybernetics epistemology, by considering yourself a part of the system you observe, the system you want to work with, you have to take the responsibility and act so as to increase the number of choices. Is this good or bad? “With this freedom of choice we are now responsible for the choice we make. For some, this freedom of choice is a gift from heaven. For others such responsibility is an unbearable burden.” (Von Foerster, 2003, p. 293) If the theory of power poses is either valid or not, I bear no responsibility, I do not need to decide, I do not need to act. But, If the theory of power posing exists in a ‘superposition of validity’, then I have the responsibility to decide, to act so as to increase the number of choices people are offered, as well as myself.

Does the replication crisis imply that psychological practice stands on clay? Not necessarily. The imperative of the replication crisis movement was that if the methodology is unsound, then the whole foundation of psychology as a discipline cannot be trusted. That’s only one reading of the story. The underlying assumption here is that psychology practitioners will read a published study, take the results as given, and attempt to apply them in the exact same way in their practice. But is this really the case? In psychology, we are not dealing with atoms or molecules. Even if a psychological experiment’s results are 100% reliable within a specific controlled context, how are they expected to apply to a different, naturalistic context? At best, the results of a social psychology experiment can provide a hint or an idea to try, and this may or may not be a problem depending on your epistemology.

Wu (2023) used simulation models to study scientific communities navigating epistemic landscapes, where solutions vary in quality, and the goal is to find the global maximum (best solution). Wu compared two strategies: the *best strategy*, where scholars adopt the best-known solution, and the *better strategy*, where they adopt any improvement over their current solution. The key finding is that communities using the better strategy outperform those using the best strategy over time. This is because the better strategy promotes diverse practices and prolonged exploration, reducing the risk of prematurely settling on suboptimal solutions. In other words, it is the “difference which makes a difference” (Bateson, 1971, p. 5), and it is the (epistemic) diversity that ensures (scientific) resilience (Capra & Luisi, 2014). While the rigid normative methodologies adopted in response to the reproducibility crisis aim at curbing negative deviance, at the same time risk stifling positive deviance, which is essential for the evolution and success of science (Phaf, 2024).

But let us revisit our driving question: To power pose or not to power pose? How to decide which answer is better? This brings us to Von Foerster’s (2003) “metaphysical postulate”: “Only those questions that are in principle undecidable, *we* can decide.” (p. 293) For Von Foerster, only undecidable questions offer true freedom of choice. Decidable questions are constrained by the framework and rules in which they are posed, leading to specific, unescapable answers. In contrast, undecidable questions, lacking such constraints, allow us the freedom to decide who we become. By increasing the number of choices, we expand the possibilities for constructing and enacting richer, more varied realities (Von Foerster, 1984). Therefore, the question should be set differently, in a way that compels us to decide, for example: Why, when, and how to power pose?

Von Foerster critiqued how societal structures, such as epistemic hierarchies and methodological positivism, are often used to avoid responsibility. He identified these structures as mechanisms for deflecting accountability by shifting decision-making power to abstract systems and scientific “algorithms”, rather than placing it on individuals bearing responsibility. This deflection, he argued, undermines the ethical imperative to act responsibly and limits the freedom of choice central to his ethical framework (Von Foerster, 2003). In first-order cybernetics (with a supposedly objective observer observing phenomena), responsibility lies exclusively within the scientific methods and tools, such as p-values or opaque AI algorithms, a kind of modern “holy scripts of science”. The human researcher or observer’s role becomes one of merely applying these methods with rigor and consistency, while maintaining a “safe” distance from the observed in order to avoid interference (e.g., the concept of randomized, double-blind, clinical trials). For Von Foerster (2003), objectivity is a device for avoiding responsibility, as “objectivity requires that the properties of the observer be left out of any descriptions of his observations. … the observer is reduced to a copying machine with the notion of responsibility successfully juggled away.” (p. 293).

### If you Desire to see, Learn How to Act

The *aesthetic imperative*: “If you desire to see, learn how to act” (Von Foerster, 1984, p. 61), emphasizes the active role of the observer in shaping their perception. This principle suggests that our ability to perceive beauty or meaning in the world is contingent on our actions and attitudes. In other words, perception is not a passive experience but an active engagement with the world (Von Foerster, 1984).

The shift from exo-science (observing from outside) to endo-science (observing from within) opens up new opportunities in scientific research (Umpleby, 2016). The emphasis on the observer’s active role encourages scholars to be more conscious of their influence on the research process and, consequently, of their impact on the people they study. If the researcher is inevitably part of the system they observe, then every research process becomes an interactive, participatory, and context-sensitive Action Research project, as understood within the qualitative research paradigm.

The concept of confluence, as defined by Gergen (2009), resonates with the principles of second-order cybernetics and offers a provocative lens for rethinking the replication crisis in psychology. Second-order cybernetics challenges the notion of an objective, detached observer by emphasizing the participatory nature of observation as an observer is inherently part of the system they study. Similarly, the theory of confluence rejects the bounded and isolated view of causality, suggesting that human actions and meanings emerge relationally, shaped by dynamic interactions. The replication crisis, often framed as a failure of methods or rigor, can be reinterpreted through this lens as a symptom of epistemological rigidity. Psychology’s positivist underpinnings rely heavily on replicable, causal models that oversimplify the complexity of human behavior, neglecting the context-dependent and relational nature of psychological phenomena: “Thus, we treat acts of aggression, altruism, and prejudice as effects, and search for an independent set of conditions that bring these about. In effect, we define the individual as fundamentally separated from the surrounding world, alone, and subject to its vicissitudes.” (Gergen, 2009, p. 51).

A shift toward confluence and second-order cybernetics invites researchers to consider relational dynamics and co-action, in order to promote research practices that embrace complexity and contextual variability. By incorporating relational epistemologies, psychological research can enrich its relevance to real-world applications: “If we wish to generate more promising futures, the major challenge is that of collaboratively creating new conditions of confluence. How can we draw from our relational histories in such a way that new and more promising confluences result?” (Gergen, 2009, p. 58).

## Concluding Thoughts: An Enabling Methodological Culture

The replication crisis in psychology reflects deeper issues than just the possibility of flawed methods or unethical practices. The study of human psychology which isolates methods from theories and assumptions can lead to oversimplification and misinterpretation of the complexity of the psychological phenomena (Valsiner, 2017). Addressing the replication crisis requires acknowledging the context sensitivity of psychological subjects and combining rigorous methodological reforms with a deeper theoretical understanding (Feest, 2024). Perhaps this is the road from experimental psychology to applied psychological practice.

In a framework of methodological eclecticism, engaging with alternatives and contrasting viewpoints while attempting to integrate them in action can be quite beneficial (Parsons, 2015). Psychologists need to be able to perform scientifically sound studies, adhering to the latest methodological developments and standards. They must also be methodologically literate enough to evaluate studies performed within a first-order cybernetics epistemology (an objective observer watching a system from a distance). Psychology practitioners working with human systems also need to be able to embrace the unfolding complexity of the here and now of a living system, to follow its process and apply their knowledge, adjusting all the time their practices and actions to the evolving situation in which they are immersed and of which they are a constituent part.

This flexible, situated, and process-oriented approach to research and practice has also been espoused in abductive approaches to knowledge generation (Brinkmann, 2014; Salvatore, 2014). Here the process of abduction connects the situation to the inquiry and to the researcher, much like feedback loops in cybernetics theory (Wiener, 1948/2019). As psychological phenomena do not pre-exist observation but rather emerge through specific intra-actions with the researcher (Sandle et al., 2024), psychologists have to “act as participants in the lives of others in order to understand them.” (Brinkmann, 2024, p. 15) Nevertheless, how researchers engage with participants during the research process matters. This engagement should be framed within an ethic of partnership as a *relational craft*, meaning that “rather than only applying abstract principles and procedural rules, researchers use ongoing, flexible judgments and creative responses to the complex challenges that arise in research situations.” (Wertz et al., 2011, p. 354).

In the misinformation age (Weatherall & O’Connor, 2024) we live in, where science is constantly challenged by pseudoscience, magical beliefs, and conspiracy theories, we need robust and sound scientific methods and practices, continuously self-correcting and ever-evolving (Sikorski & Andreoletti, 2023) within a first-order cybernetics framework. However, we should not sweep under the rug the inevitable epistemological assumptions we make during the process. On the contrary, epistemological assumptions should always be made visible and discussed with an open minded attitude: “if I don’t see I am blind, I am blind; but if I see I am blind, I see” (Von Foerster, 1984, p. 43). The current normative methodologies, with their focus on preregistration and statistical rigor, operate under a linear conception of science, assuming an objective observer that observes the whole process (Phaf, 2024). But, the replication crisis is not just a statistical problem; it is a broader issue requiring significant backstage philosophical and epistemological work to ensure science’s reliability and progress (Gervais, 2021; Malich & Rehmann-Sutter, 2022; Romero, 2019). Such an approach will make the scientific method more trustworthy and helpful for individuals and communities.

From Feyerabend’s (1975) seminal book *Against the method* to the methodological reformation movement for experimental psychology and the resulted scientific debate and crisis (Derksen & Field, 2022), there is a great epistemological distance. A scientific community that remains open to more diverse perspectives and information gains an epistemic advantage (Wu, 2023). This diversity allows the research community to explore a broader range of potential theories (Gervais, 2021). Successful science requires space for deviant ideas that challenge existing norms, as historically, groundbreaking scientific innovations often arose from unconventional hypotheses that initially appeared implausible (Phaf, 2024). However, too much diversity or prolonged exploration of inferior theories can be harmful, delaying critical scientific progress, especially in clinical practice (Wu & O’Connor, 2023). How can the scientific community balance between the two? Here comes the need for epistemological superposition to avoid a binary answer. Phaf (2024) argues for an evolutionary conception of science, where competing theories are allowed to co-exist and evolve through trial and error. We need to move away from a toxic scientific ecosystem (Gervais, 2021) toward an enabling methodological culture. We don’t have to fear epistemological differences; rather, we need to learn from them.

## Data Availability

No datasets were generated or analysed during the current study.

## Footnotes

1 Retrieved from: https://faculty.haas.berkeley.edu/dana_carney/pdf_My%20position%20on%20power%20poses.pdf.
